# Discoid Lupus Erythematosus Successfully Treated With Anifrolumab

**DOI:** 10.7759/cureus.108173

**Published:** 2026-05-03

**Authors:** Jake Breimann, Shayan Waseh, Sylvia Hsu

**Affiliations:** 1 Dermatology, Temple University Lewis Katz School of Medicine, Philadelphia, USA; 2 Dermatology, Temple University Hospital, Philadelphia, USA

**Keywords:** anifrolumab, chronic cutaneous lupus erythematosus, discoid lupus erythematosus (dle), saphnelo, type 1 interferon

## Abstract

Discoid lupus erythematosus (DLE) is a chronic autoimmune condition characterized by scarring, dyspigmentation, and atrophy, most commonly affecting sun-exposed areas. Standard treatments such as corticosteroids, antimalarials, and immunosuppressants are often inadequate, and refractory cases can result in significant cosmetic and psychological morbidity. We present the case of a 27-year-old woman with recalcitrant DLE involving the face, scalp, arms, and hands. She failed to achieve symptomatic or cosmetic improvement with prednisone, hydroxychloroquine, or thalidomide. Due to persistent disease activity, she was initiated on anifrolumab, a monoclonal antibody targeting the type I interferon receptor. Within two months, she reported marked improvement in symptoms, and physical examination showed residual hyperpigmented macules without new lesions. This case highlights the potential utility of anifrolumab as a targeted therapeutic option in DLE and underscores the need for further studies evaluating its long-term safety and efficacy in cutaneous lupus.

## Introduction

Discoid lupus erythematosus (DLE) is a disfiguring chronic autoimmune disease characterized initially by erythematous papules or plaques on sun-exposed areas, which often proceed to scarring, atrophy, dyspigmentation, and permanent alopecia. Although a variety of treatments, including topical steroid therapy, antimalarial therapy, thalidomide, and systemic immunosuppressants, have been traditionally employed, many patients continue to suffer from refractory disease [1]. Early and effective treatment has the potential for prevention of long-term sequelae, but scarring remains a common outcome with profound psychological morbidity [2]. Here, we report a case of DLE demonstrating substantial improvement with anifrolumab.

## Case presentation

**Figure 1:**
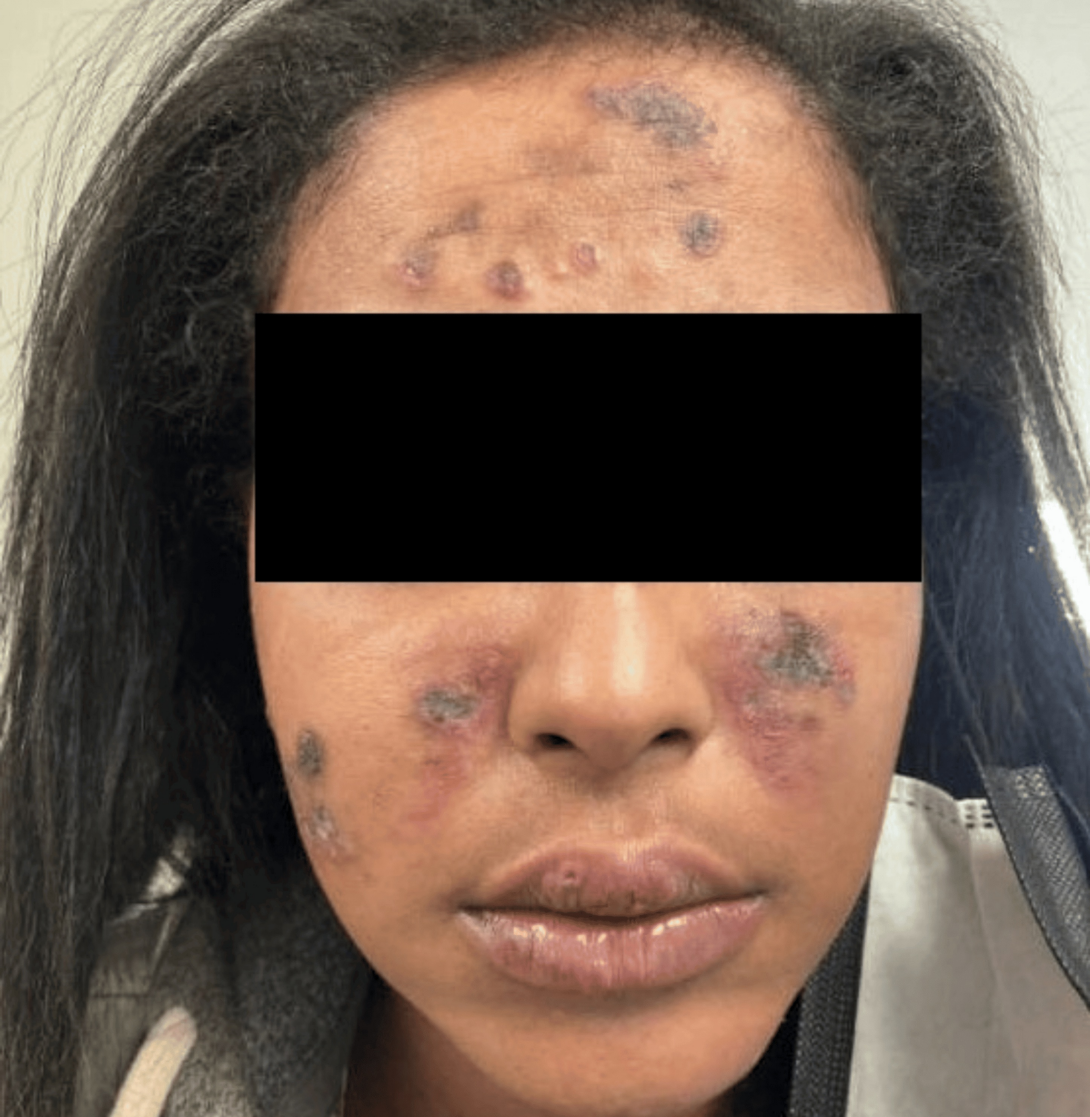
Baseline presentation of active discoid lupus erythematosus prior to anifrolumab Physical exam notable for erythematous, crusted plaques of the forehead and cheeks.

**Figure 2:**
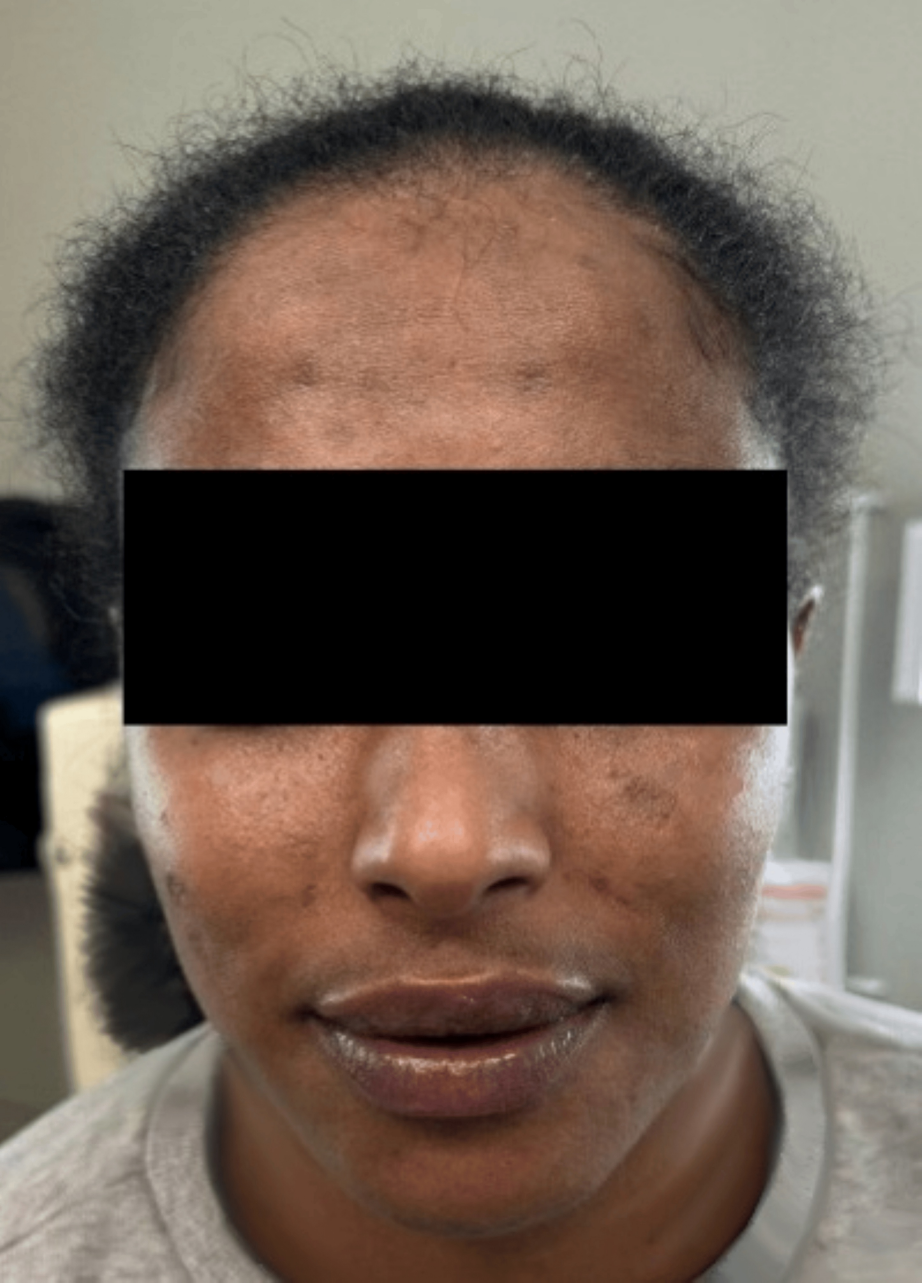
Clinical improvement of discoid lupus erythematosus after two months of anifrolumab Physical exam notable for reduced peripheral erythema and plaque formation with residual post-inflammatory hyperpigmentation.

A 27-year-old woman with an unremarkable past medical history presented to the dermatology clinic with two months of hyperpigmented, erythematous, pruritic, and painful plaques on her face (Figure 1), scalp, arms, and hands. Given the photodistribution of her rash and clinical presentation, she was diagnosed with DLE without biopsy and referred to rheumatology for further evaluation of systemic lupus erythematosus (SLE). Serologic testing revealed a positive ANA at a 1:320 titer, low C3, positive anti-RNP, with negative anti-dsDNA, anti-Smith, RF, anti-CCP, C4, and SSA/SSB antibodies. Testing results are listed in Table 1. Upon rheumatology consultation, she reported knee synovitis but lacked systemic features. Systemic Lupus International Collaborating Clinics (SLICC) criteria were met for diagnosis of SLE with cutaneous lesions consistent with DLE, synovitis of the knees, +ANA, and a low C3. Over a two-year period, she tried hydroxychloroquine 200 mg twice per day, various topical steroids, and prednisone 60 mg daily taper over two months without cosmetic or symptomatic improvement of her DLE. Due to her refractory disease, she was then trialed on thalidomide 50 mg nightly for eight months with minor improvement, but her course was complicated by peripheral sensory neuropathy. In light of newly emerging evidence for the use of anifrolumab for refractory discoid lesions, the patient was subsequently started on anifrolumab 300 mg intravenous infusions monthly with continuation of hydroxychloroquine 200 mg twice per day and clobetasol spot treatments as necessary. At the initial two-month follow-up, the patient reported significant improvement in her symptoms. On physical exam, small, residual hyperpigmented macules and patches remained on the face without additional flares (Figure 2).

**Table 1:** Summary of the patient's serological results with corresponding laboratory reference ranges ANA: Anti-nuclear antibody, Anti-DsDNA: Anti-double-stranded DNA, Anti-RNP: Anti-ribonucleoprotein, Anti-SSA: Anti-Sjögren's-syndrome-related antigen A, Anti-SSB: Anti-Sjögren's-syndrome-related antigen B, C3: Complement component 3, C4: Complement component 4, Anti-CCP: Anti-cyclic citrullinated peptide, RF: Rheumatoid factor

Serological Marker	Value	Reference Range
ANA	1:320	<1:40
Anti-DsDNA	2 IU/mL	≤4.0 IU/mL
Anti-Smith	<1.0	<1.0
Anti-RNP	Positive (no reflex to titer)	<1.0
Anti-SSA	<1.0	<1.0
Anti-SSB	<1.0	<1.0
C3	62 mg/dL	90-180 mg/dL
C4	16 mg/dL	10-40 mg/dL
Anti-CCP	<16 U	<20 U
RF	<10	0-35 IU/mL

## Discussion

Anifrolumab was approved by the FDA in July of 2021 for the treatment of moderate-to-severe SLE patients who are currently receiving standard therapy including one or a combination of oral corticosteroids, antimalarials, and/or immunosuppressants [3]. Recently, new studies using anifrolumab as an off-label agent to treat cutaneous lupus, including discoid, have demonstrated promising results [4,5]. In a 10-patient case series of refractory DLE, anifrolumab treatment was associated with a mean 65.1% reduction in Cutaneous Lupus Erythematosus Disease Area and Severity Index (CLASI) activity scores within eight weeks. In addition, a four-patient case series demonstrated rapid improvement across various cutaneous lupus subtypes, including generalized DLE with CLASI, an improvement from 40 to 8 within two months. In conjunction with this newly emerging data, this case report provides further evidence for the use of anifrolumab in the treatment of refractory DLE. While anifrolumab is generally well tolerated, it carries the risk for side effects including serious infection and possible increase in risk for malignancy, as well as a risk for hypersensitivity reactions including anaphylaxis.

The clinical responses observed across these cases may be mechanistically explained by the role of type I interferon signaling in DLE. Anifrolumab works by targeting type I interferon receptor subunit 1 (IFNAR1), which inhibits the activation of the type I interferon signaling pathway. This pathway is produced in excess by plasmacytoid dendritic cells, which promote a pro-inflammatory cascade, and these cells are increased in DLE lesions [6]. In addition, type I IFNs are also observed to stimulate the expression of TNF-related apoptosis-inducing ligand, apoptosis receptor CD95, and other cytotoxic proteins involved in apoptotic pathways, contributing to the accumulation of cell debris and subsequent autoimmune inflammation [7]. By blocking this pathway, anifrolumab may help decrease inflammatory activity, decrease apoptosis-related tissue damage, and serve as a targeted treatment for patients with refractory DLE.

## Conclusions

This case contributes to emerging evidence supporting the use of anifrolumab in managing DLE that is resistant to conventional therapies. By inhibiting type I interferon signaling, anifrolumab may help interrupt the inflammatory cascade central to DLE pathogenesis. The patient’s rapid and meaningful improvement underscores the therapeutic promise of interferon blockade in select cases of refractory disease. While encouraging, these findings highlight the need for additional studies to better define the long-term efficacy, safety profile, and appropriate clinical context for anifrolumab in DLE. Ongoing research into cytokine-targeted treatments may help refine management strategies and improve outcomes in patients affected by this chronic and potentially disfiguring condition.
